# Bringing together coproduction and community participatory research approaches: Using first person reflective narrative to explore coproduction and community involvement in mental health research

**DOI:** 10.1111/hex.12908

**Published:** 2019-06-11

**Authors:** Colin King, Steve Gillard

**Affiliations:** ^1^ St George’s, University of London London UK

**Keywords:** community mental health services, community participation, community‐based participatory research, consumer involvement, mental health, patient involvement, research methodology

## Abstract

**Background:**

A growing literature explores the coproduction of research knowledge. Barriers to coproduction in mental health research have been identified, especially for the people from marginalized communities. There is an established body of participatory research that has potential to inform coproduction in mental health research.

**Objectives:**

To explore and articulate how learning from community participatory approaches to research enable barriers to knowledge coproduction to be overcome in mental health research.

**Setting:**

An evaluation of a primary care mental health service, led by an experienced survivor researcher, supported by a health service researcher and involving a team of community co‐researchers.

**Design:**

Cycles of reflective writing (first‐person narrative) by the authors, and feedback from the co‐researcher team, on their experiences of undertaking the evaluation were used to explore the ways in which community actors, including those from marginalized communities, might be meaningfully involved in producing research knowledge about mental health services.

**Results:**

A space was created where community co‐researchers, including those from traditionally marginalized communities, felt safe and empowered to move beyond essentialized “service user” identities and bring a range of skills and expertise to the evaluation. There was meaningful rebalancing of power between traditional university and community roles, although the issues around leadership remained complex and more could be done to explore how our different experiences of race and mental health shape the research we do.

**Conclusions:**

Potential was demonstrated for participatory research approaches to inform coproduction of knowledge in mental health research that fully reflects the diversity of identity and experience.

## 1 BACKGROUND

A growing literature explores patient and public involvement in health and social care research, including in the field of mental health. Some of this literature focuses on a radical survivor‐ and service user‐led research, and the related field of “mad studies.”^1^ Other writing explores the role of “service user researchers”—researchers who bring both academic training and lived experience of using mental health services—working as part of conventional clinical academic teams.^2^ Thinking about the “coproduction of knowledge,” borrowed from the public engagement in science field, has begun to influence this work. Coproduction suggests a move away from academics and academic institutions as the sole arbiters of what constitutes scientific knowledge, introducing a social accountability to research whereby an “expert laity” contributes to shaping the research process in a less hierarchical, more distributed structure.^3^ This was demonstrated in a mental health research project undertaken by a interdisciplinary team including researchers working from a perspective informed by their personal experiences of using mental health services. Coproduction was described as: high‐value research decision making distributed across the team; an interpretive approach understood in terms of team members’ identities; methodological flexibility in the research process; critical reflection on how the research was done; reporting on how knowledge was produced.^2^ The importance of quality of dialogue in the research team to support coproduction, especially where there might be differences of views about what constitutes valid knowledge, has been noted.^4^, ^5^ The UK body that supports patient and public involvement in health‐care research identifies the key principles of coproduction as: sharing of power, including the perspectives and skills, and respecting and valuing the knowledge of all those involved; reciprocity where everyone benefits from working together; an emphasis on building and maintaining relationships.^6^

There are barriers to realizing this coproduction in practice. The requirement of most universities for researchers to be graduates can limit access, with involvement in research for many limited to an advisory capacity. Issues of resources, methodological hierarchies and priorities for academic publication can also constrain opportunities,^7^ while the conspicuous absence of service user–led or survivor‐led research from the mental health research funding agenda is also noted.^8^ It has been suggested that processes of academic peer review function to privilege some forms of knowledge over others, acting as an “epistemological protectionism” absolving academics of the need to engage more widely.^9^ In addition, it has been suggested that marginalization that exists in public institutions—especially with regard to race and ethnicity—is perpetuated in mental health research. Beresford and Rose^7^ note how user‐controlled and survivor research focusing on and involving Black and minority ethnic (BaME) mental health service users and survivors is thin on the ground compared to that involving their white counterparts. Kalathil^10^ has suggested that hierarchies of power that persist in mental health services are replicated in user involvement spaces where professionals maintain a hold on the role of expert and control agendas, and that these spaces are further disempowering for people from racialized groups as cultural and racial identities are silenced as a result of failure to openly discuss the discrimination that characterizes services. Indeed, the idea that academic practice more generally mirrors the exclusions found in society has long been maintained, with, for example, Ladner^11^ stating that mainstream or “White” sociology has worked to uphold the status quo in race relations in the USA through largely denying the differing historical conditions that underpin cultural experience. More recently, King^12^ notes the relevance of Fanon's^13^ exploration of the “white mask”—assumed by people of colour as a way of becoming culturally invisible and thereby staying safe in racially hostile environments—to the experiences of psychiatry of men of Black African cultural heritage. As such, attempts to coproduce research about mental health services are at risk of reproducing, or at least struggling to challenge, the marginalization of communities who, for one reason or another, find themselves excluded from, or silenced within those services.

Elsewhere, research in development studies has noted the need to create boundary spaces that enable people from different social worlds—academic and community actors—to interact, make visible their different thoughts styles and learn together.^14^ Reflecting on these endeavours, Durose et al^15^ note the potential for participatory research traditions to expand our thinking about coproduction and move coproduction in research from the merely dialogical to the transformative.^16^ Although not a single method or approach, participatory research tends to focus on “processes of sequential reflection and action, carried out with and by local people rather than on them” (p. 1667).^17^ This is differentiated from more conventional research by a re‐alignment of power within research relationships and a recognized need to integrate local knowledge and experience into the research process. The key features of participatory research have been characterized as: a democratizing approach with respect to supporting the participation of under‐privileged demographic groups; creation of a “safe space” in which people can communicate with openness and trust; and community participants actively taking on a “co‐researcher” role that empowers them to use the knowledge they bring to the research.^18^

Community‐Based Participatory Research (CBPR) has been offered as an approach to enhancing the “cultural competence” of health and social care research,^19^ in particular as an approach to health disparities research that “embeds the cultural context and beliefs of community researchers into the research study” (p. 214).^20^ Mosavel et al,^21^ in research on cervical cancer in South Africa, note the potential of participatory research to address the “silent dynamics of race” and its powerful and unspoken role in reinforcing Euro‐centric methodological frameworks. Mayan and Daum^22^ note the potential for tensions and conflict to arise as relationships—between community members, academics and service providers—become blurred by the participatory process, while Stoecker^23^ warns against participatory research that invites people into the process of producing knowledge—for example being involved in collecting data—without “credentialed” researchers giving up power over deciding how that knowledge is to be produced.

Interestingly, Sweeney^24^ acknowledges the influence of participatory research on shaping service user research but notes the tendency of participatory approaches to focus on the micro—the experiences of individuals—while neglecting the macro, political level, suggesting that survivor‐ or user‐controlled research offers a more emancipatory potential by ensuring that the lead researcher is necessarily also a “community member.” Similarly, Russo cautions that power relationships are not equalized through participation alone, and that community leadership is required to ensure that the standpoint of the research is embedded within the experience and priorities of the community.^25^

### 1.1 Aims

This paper explores and articulates the ways in which learning from community participatory approaches to research enables barriers to knowledge coproduction, as identified above, to be overcome in mental health research. We ask whether a participatory‐informed approach to coproducing a mental health research project manages to: (a) create spaces in which community actors, including those from habitually marginalized communities, can meaningfully contribute to the production of research knowledge; (b) address power imbalances between traditional academic and service user and community researchers, including through service user/survivor leadership. We describe and critically explore an evaluation of a primary care mental health service in England as a means of considering those questions.

### 1.2 The evaluation

The evaluation was commissioned by a locality state health service funding body, at the behest of their service user and carer advisory group, from a mental health research team at a local university. The research team had undertaken a previous service evaluation in the area,^26^incorporating elements of participatory research and survivor leadership in the process, and the new evaluation was commissioned to employ a similar approach. The evaluation was led by an experienced, university‐based survivor researcher, Colin, with the support and guidance of a health services researcher, Steve. Colin, a Black British man, was recruited specifically to lead the project, having previously completed a PhD and a number of pieces of independent research in the field of race and mental health from a survivor perspective. Steve, a White British man, had been working in the university for several years, leading mental health research that supported researchers with personal experiences of mental distress as integral members of research teams, designing and delivering research.

Colin was given the report from the earlier evaluation as a starting point but was free to take decisions about how best to undertake the new evaluation. Steve was Colin's line manager, providing regular supervision and monitoring progress in delivering the evaluation through a project plan regularly updated by Colin. Steve held responsibility for delivering the final report to the commissioners.

Colin and Steve decided that six co‐researchers would be recruited to work alongside Colin to undertake the evaluation, as well as six Lived Experience Advisory Panel (LEAP) members, to provide oversight and advice to the evaluation, from an experiential perspective, at periodic meetings. Co‐researchers and LEAP members were recruited both through service user groups in the local community and through wider service user researcher networks. A total of eleven people were recruited, seven from the local community and four from wider networks, including eight women and three men. Four people were Black or Black British, four were White or White British, two were Asian or Asian British, and one was Other Ethnic Group (official UK census ethnicity categories). While recruited to different roles, in practice all eleven met together at all times and all took on the co‐researcher role. This was a decision taken by Colin, with the agreement of Steve and the co‐researchers early in the project.

The evaluation comprised an online and postal survey sent to a systematic sample of people who had recently made use of the primary care mental health service, focus groups, and face‐to‐face and telephone interviews with a subsample of survey respondents. Colin led on recruiting co‐researchers, coordinating team meetings, developing and finalizing the evaluation process, supporting co‐researchers with evaluation tasks and writing up the evaluation report. Steve co‐facilitated team meetings, provided methodological advice to Colin and the co‐researcher team and assisted in writing up the evaluation report. Co‐researchers were involved in developing survey, interview and focus group tools, interviewing and conducting focus groups, analysing survey, interview and focus group data, writing up sections of the evaluation report and presenting findings at the evaluation report launch.

## 2 METHODS

We use first‐person reflective narrative of the evaluation process as a way of exploring the methodological approach. Colin and Steve each produced, independently, written first‐person accounts of their experiences of setting up and carrying out the evaluation shortly after the evaluation was completed. These accounts were then iteratively co‐edited by the authors, in the form they appear below, with Steve undertaking an initial edit organizing the narratives under subheadings relating to stages of the evaluation. Rounds of editing took place through face‐to‐face and email discussion between Colin and Steve, selecting narrative that responded to the specific questions identified above. Our shared writing and re‐writing was integral to our method.^27^, ^28^

All members of the co‐researcher team completed a short, written questionnaire reflecting on their experiences of the evaluation, again shortly after the evaluation was completed. The questionnaire asked:

1. What did you expect to be doing as part of the evaluation before we started?
2. How would you describe your involvement in the evaluation?
3. What went well (what did you enjoy doing, where do you think your involvement made a difference, etc)?
4. What might we do better in future evaluations of this sort?

Colin and Steve selected responses to the questionnaire to further illustrate the process and both contributed to commentary around those responses, combined below. Our writing was shared with the co‐researcher team at first‐draft stage. Written and verbal feedback from co‐researchers on the first draft was generally approving. Two co‐researchers made suggestions for shortening and focusing the paper onto specific aspects of the evaluation process which we incorporated into the final version.

### 2.1 Findings

#### 2.1.1 Starting out

Colin: I walk up the stairs, into a room of four white interviewers, one a white male project manager [Steve], and three white female researchers with a lived experience of mental health. The position, survivor researcher to train and lead a user group to undertake an evaluation project of a local primary care mental health service. I sweat, pause, internalizing my inferiority and incompetence, my black skin is concealed by my white mask. I hear my answers as fragmented and incoherent in my deference. I am shown around the research department, white, freshly painted walls, groups of individuals locked behind heavy brown doors, few people of colour. On the first day, I collect my identification card, keys to the research room with three white female researchers on one side, myself and an Asian male researcher on the other side of the room. I am haunted by the fluency of the research language, the depth of the coded language used and the fear of the challenge of carrying out the research into a mental health project I know nothing about.

Steve: When we interviewed for the service user researcher/project coordinator post for this project we wanted someone who demonstrated understanding and experience of both the opportunities and challenges offered by service user involvement in research, and also the wider issues of engaging communities in research. Colin was extremely articulate on all counts, both in his job application and in the interview. Colin was also our only male applicant and our only Black applicant. The evaluation project, as it was commissioned, did not have a specific focus on race and ethnicity and so this was not a particular consideration in the appointment process. However, while we had recently employed a male Asian researcher, over the years the majority of our team has been female and White. Colin's appointment was a welcome opportunity to bring awareness and critical thinking around race, ethnicity, research and mental health to our wider team as well as to this project.

#### 2.1.2 Recruiting the co‐researcher team

Colin: The recruitment took place through an existing service user research advisory group based at the research department and the research team's wider networks. I phoned, interviewed, and talked to a variety of potential co‐researchers and LEAP panel members. All eleven people I spoke to had the potential to be either co‐researchers or LEAP members, with the balance of men and women, Black, White and Asian, reflecting the diversity of their social worlds. In assigning people to roles it felt as though their skills were being elevated above their ‘lived experience’ of mental distress. I also felt that the interviews, the interaction and the talk on a one‐to‐one level as I met with people provided the foundation for coproduction at the micro level.

Steve: I largely left this to Colin to do and what struck me was his very hands on, person‐focused style. In the department we had established quite a formal way of recruiting co‐researchers and research advisors to ensure the process was equitable, and because we felt that doing things formally indicated the value of the appointment. Colin didn't neglect those values but his approach was much more relational, to speak to people as often as necessary on the phone and to meet them face‐to‐face when and where that worked for people. It was as though Colin was building those relationships from the outset, getting to know people as they got to know him.

#### 2.1.3 Coproducing the research

Colin: Methodologically I inherited a very structured evaluation framework, based on the team's earlier project. The challenge for coproduction in this context would be to continually reflect on and balance the demands of the research as it was commissioned and the ideas and interests brought by the LEAP and co‐researchers. During the monthly planning meetings the team revealed a range of research, project management and reporting skills. In this context credibility was given primarily to people's skills as opposed to their experiences of mental health. What became essential was creating an environment of equality, equity and empowerment. The team were always welcomed by the non‐discriminatory attitude of the department administrator who responded to them with a human, personalized dignity, without the patronizing tone often used almost to caricature mental health service users. People were encouraged to lose that differential (mental illness) aspect of their identity, and to perform from the center of their diverse identities as they were invited to engage critically with the evaluation process.

What emerged was a liberation from prescribed roles as boundaries were broken and what people did was matched with their interests and abilities. The focus was on the social (of who we are) and led to a dismantling of the demarcation of the LEAP and co‐researcher roles, rich dialogue in the team and a flexible approach to the methodological challenges of the evaluation. Tasks in terms of the design of the survey, interview and focus groups were open to all team members, the distinct skills of the eleven people involved emerged and their experiences of mental distress became secondary.

Steve: Although based closely on our original evaluation we also agreed that the structure of the evaluation would not be fixed and could be developed with the co‐researchers and the LEAP at the outset of the project. I attended all of the co‐researcher meetings, helped facilitate activities and provided advice on methodological options when asked or when I felt it was helpful to suggest options. Colin led the meetings and I was struck by his inclusive and engaging way of enabling people to get to know each other, his self‐deprecating manner, use of humour and the sense of enjoyment he brought to the project. From the outset he made clear to people that they would be doing the evaluation, not him, and people seemed empowered by the approach. Almost from the outset ideas about how we might undertake the evaluation flowed around the table. The experience was liberating for me as in most projects, as lead investigator, I would feel the responsibility for methodological decisions. Here I had a freedom to respond creatively to the ideas coming from the team and to explore ways in which we could realise their aspirations for the project.

A case in point was the meeting held to plan the process of analysing interview and focus group data. Having conducted and transcribed the interviews and focus groups, the team was already sharing, around the table, the themes that might constitute an analysis. Not wanting to lose that momentum and focus, we decided between us to improvise an analytical process whereby each person first wrote down the key messages emerging from the interviews they had conducted, then shared them verbally. We then refined, through discussion, a final set of themes that captured and made sense of their collective response to the accounts they had elicited.

### 2.2 What the co‐researchers had to say

Individual expectations of the evaluation included acquiring knowledge and developing research skills. On the level of involvement, co‐researchers commented that:

[I was] pleasantly surprised at the depth of our involvement.

I have appreciated the autonomy and high level of involvement that Colin has given us in the evaluation process. In doing this, he has demonstrated his faith in our ability and respect for our experiences as peer researchers … I have been left feeling that I have made a worthwhile contribution to the research process and that I might be capable of running a similar project myself …

Early in the project co‐researchers did ask for clarification of the co‐researcher and LEAP roles, but through discussion supported Colin's suggestion that all would be actively involved as co‐researchers. Co‐researchers reported how cohesion developed in a mixed team:

The process of collaborating as a team of … people with lived experience and as professionals worked well. We generated ideas and spurred each other on. We also made alliances and friendships … where we supported each other within and outside meetings in regard to certain points of work, or for emotional support. This was important for the rapport and cohesion of the team in sustaining motivation and morale.

I found the mix of the team with lived experience peers, researchers and professionals was comfortable. There was a mix of gender, age and ethnicity too to get a wider view … the team had varied skills and these were encouraged.

This link between the project feeling comfortable and co‐researchers feeling enabled to bring their skills and expertise to the evaluation was also noted by another co‐researcher:

I felt I could comfortably share my expertise of how we could proceed with the evaluation by giving ideas like compiling surveys and the type of questions we needed to frame.

Mistakes were made, with some co‐researchers feeling that the university researchers’ communication could have been better; some co‐researchers were inadvertently left off email lists and short notice given for some meetings making it difficult for some people to plan their time in advance. One co‐researcher noted:

For future projects try to prevent communication breakdowns as they caused missed opportunities and disappointment.

One member of the team felt that there should have been more preparation for interviewing, with another finding some of the activities too short and intense. One member of the team noted that the time taken to do pieces of work sometimes exceeded the time allowed, resulting in some co‐researchers contributing on a voluntary basis:

[This did] not show high appreciation or value for co‐researchers. There is no parity of esteem with the professional researchers.

Nonetheless, co‐researchers did feel that their role in shaping and undertaking the evaluation was enabling for participants and productive of good data:

I felt like my involvement in the focus group was particularly helpful to the … clients involved, as they really got to talk and express their views about the service. I think they appreciated that we, as people with lived experience of mental health issues, could relate to their experiences and we made them feel confident to talk about their feelings and views …

I enjoyed doing interviews, I hope my genuineness, active listening, reflecting skills helped the participants to be more open about their opinions and experiences.

## 3 DISCUSSION

In this paper, we explored the potential for community participatory approaches to address barriers to knowledge coproduction that have been identified in mental health research. We reflect on our narratives to consider the specific challenges posed to us by the literature.

### 3.1 Creating spaces for coproduction, addressing marginalization

Colin wrote above about putting on his white mask^13^ in order to feel safe, as a Black man, when he first entered the university environment. Colin's approach, as he guided and supported the co‐researchers, was predicated on creating a space in the meeting room—once the ubiquitous white walls and perpetually closed doors had been negotiated—wherein co‐researchers felt safe in expressing all aspects of their identity,^18^ and not just in attempting to perform as “researchers.” Colin acknowledges the important role played by the department's administrator in helping to create that welcoming space. Co‐researchers were initially “surprised” at the “faith” shown, but felt “encouraged” and became “comfortable” in fully expressing themselves and contributing their skills and expertise. Once our co‐researchers experienced, through interaction, a sense of empowerment, they felt able to contribute fully to the evaluation process.^3^ Not without mistakes, we managed to create the safe “boundary space”^14^ in which that open communication was possible.^18^

As noted, our project was not specifically about race, but we strived to recruit a co‐researcher team from across the diversity of our local community through Colin's very personal approach. Colin skilfully circumvented some of the rigid processes that characterize entry to academia, identified by both survivor researchers^7^ and writers on race^11^ as restricting access to people from marginalized communities. However, we note the words of caution from Stoecker^23^ and Sweeney^24^ against focusing on the dynamics within the team at the expense of the wider, political rationale for the participatory approach, in this case, ensuring that habitually marginalized voices were not just present, but also instrumental in the evaluation process. We also note how remaining silent about race as we work together influences the way we do research.^20^, ^21^ Colin and Steve began to explore their different personal experiences of race, and advantage or disadvantage, in relation to mental health and research, reflecting other work that seeks to understand mental health from the perspective of what it means to be White, as well as Black.^29^ However, while our conversations as a wider team were certainly about ethnicity and inclusion—both within the team and among the evaluation participants—we did not, by and large, extend those more challenging conversations about personal experiences of race in relation to mental health to the co‐researcher team. Perhaps we missed an opportunity, in our evaluation, to explore, more explicitly, issues of access, experience and outcomes in the primary care mental health service in relation to race and equality.

### 3.2 Addressing power imbalances, realizing service user/survivor leadership

We suggest that we achieved some measure of success in addressing power imbalances traditionally inherent between university and community actors. Mayan and Daan^22^ refer to a muddling of concepts whereby co‐researchers are judged firstly in relation to their lived experience, with their research skills and attributes seen as secondary, whereas, more hopefully, Goffman^30^ envisages a merging of “front and back stages” whereby people move from the stereotypical roles allocated to them as “mental health patient” and begin to perform their whole self. We feel that we managed to move beyond an essentializing “service user” identity for our co‐researchers, foregrounding the range of skills and expertise that the team brought to the evaluation while recognizing the importance of the “lived experience” that they also embodied. While co‐researchers were appointed because of their experiences of mental distress, we managed to provide an environment in which the emergence of an identity as “researcher” was possible, with co‐researchers negotiating their role and appreciating the range of skills they were able to put into practice.

We suggest our sharing of decision‐making responsibility and flexibility of research methods^2^—for example in the analysis of interview and focus group data—was also indicative of a measure of rebalancing of power in our evaluation. Cornwall and Jewkes^17^ speak of the importance of being alive to “sequential reflection” and use of “innovative adaptive methods” in order that the democratization of the research process is not stifled by the university researchers’ better wisdom. In a sense, we gave up a measure of power over the research process to enable our co‐researchers to put their insight and expertise into practice.^23^

We might argue that it was our efforts to incorporate a community participatory approach that enabled us to exercise the shift of power called for more generally by survivor researchers and mad studies scholars.^1^ Sweeney^24^ and Russo^25^ note the importance of community leadership in research to ensure that any sharing of power is more than superficial. Colin was specifically appointed to lead the evaluation from a survivor perspective, but we also note that Colin was accountable to Steve, a more senior researcher in the university who held responsibility for delivering the evaluation. Colin did demonstrate real leadership over appointing and shaping the roles of the community co‐researchers, and in developing an empowering culture of practice within the team, while Steve exerted influence through making suggestions about methodological processes in response to the ideas put forward by the team. We also see above that Colin felt more bound to the framework inherited from the previous evaluation than Steve had intended because of the need to live up to methodological expectations and deliver what was required by the funder. We reflect that a more radical service user or survivor leadership^1^—full control over the evaluation process—was not realized here, but we do suggest that, as a survivor researcher, Colin exercised considerable leadership over the evaluation process and was able, as a result, to ensure that the priorities and processes of the evaluation were shaped to a meaningful extent by our community co‐researchers.^18^

## 4 CONCLUSION

We conclude that our hybrid participatory and coproduction approach to evaluation was characterized by our successfully creating—mistakes notwithstanding—a safe space in which our different and complex skills and expertise as a team were productively brought together. We identify a “productive paradox” at play here; our co‐researchers were welcomed into the university and made to feel comfortable enough that they could bring the whole of their self to the evaluation process, rather than having either to perform as a researcher to be accepted or to conform to a prescribed “service user” identity. As such, they were enabled to contribute to the evaluation process a whole range of skills, experiences and expertise that reflected their complex identities. Yet, while our co‐researcher team reflected the diversity of our local community, we perhaps missed an opportunity to engage fully in the more difficult work of locating our evaluation in the historical and political context of race and mental health that might be advocated by Ladner.^11^

Issues of leadership remained complex, with Colin and Steve's relationship in part defined by the expectations and terms of the commissioned project and the university context. But we note important leadership functions demonstrated by Colin, first walking the ground himself as a survivor researcher and a Black man, preparing the space in the university in which to welcome the community co‐researchers and then empowering them through demonstrating his faith in their ability to deliver the project and in the range of skills and experiences they brought. We think we shifted some of the traditional imbalance of power between university and community researchers noted in the literature; there was both giving up and sharing of power over decisions and processes. That felt meaningful—this was more than us just getting on well as a team—and in our efforts to coproduce knowledge about mental health services, we hopefully moved beyond the merely dialogic,^16^ offering an approach to coproduction grounded in community and the full complexity of all of our identities.

## CONFLICT OF INTEREST

The authors have no conflicts of interest to declare.

## DATA SHARING STATEMENT

The data that support the findings of this study are available from the corresponding author upon reasonable request.
